# Collective Wisdom in Faculty Development for Competency-Based Medical Education: A Needs Assessment and Survey

**DOI:** 10.15694/mep.2021.000152.1

**Published:** 2021-06-03

**Authors:** Giovanna Sirianni, Shantel Walcott, Susan Glover Takahashi

**Affiliations:** 1 Sunnybrook Health Sciences Centre; 2 Postgraduate Medical Education; 3 Postgraduate Medical Education and Department of Family & Community Medicine

**Keywords:** Competency-Based Medical Education, CBME, Competence by Design, CBD, Faculty Development, Medical Education, Residency, Strategic Planning, Feedback, Coaching

## Abstract

This article was migrated. The article was marked as recommended.

**Background:** As programs transition to competency-based medical education (CBME), faculty development (FD) will be a key component of supporting successful implementation.

**Methods:** Faculty at the University of Toronto (UofT) with leadership roles in residency education were invited to complete an online survey to explore their experiences with FD for CBME. Descriptive statistics were collected. Results were analyzed using thematic, frequency and comparative analyses between respondent subgroups to identify trends and theme categories relevant to the perceived most effective FD activities, most helpful FD topics as well as program/department needs for future FD initiatives.

**Results:** The overall survey response rate was 44.6%. The most effective FD activity identified by survey respondents was a small group format. Perceived top FD topics included implementing CBME, assessment tools, feedback and coaching along with competence committees. The majority of programs felt that the ideal timing for CBME implementation was 6-12 months prior to implementation. The main perceived barrier to FD was lack of time amongst faculty.

**Conclusions:** This data helped drive FD at UofT by supporting strategic planning for implementing competency based curricular reforms. The results have also informed the need for additional resources and enable focused FD on barriers and needs.

## Introduction

Across Canada, undergraduate and postgraduate medical training programs are undergoing significant change as they transition to competency-based medical education (CBME) models. In postgraduate medical education (PGME), the College of Family Physicians of Canada (CFPC) has implemented the Triple C Curriculum which reflects the key elements of its competency-based curriculum: comprehensiveness, continuity in learning experiences as well as patient experiences and centered in the family medicine domain ( Iglar, Whitehead and Glover-Takahashi, 2013; Gutkin and Triple, 2011). The Royal College of Physicians and Surgeons of Canada (RCPSC) is utilizing a stepwise approach to CBME implementation named Competence by Design (CBD) ( Harris and Frank, 2014). Curricular modifications are well underway across universities including the University of Toronto (UofT); however, as programs transition to an outcomes-based approach to curriculum, assessment and evaluation, there is clear evidence that faculty feel unprepared for the tasks necessary to implement successful CBME ( Harris and Frank, 2014). A recent scoping review on the landscape of FD in CBME has also shown that there is a paucity of literature in this area, despite the acknowledged importance of FD for CBME implementation and success ( Sirianni, Takahashi and Myers, 2020).

It has been suggested that to ensure an effective CBME program there is a need for frequent, longitudinal assessments of learners, direct observation and narrative feedback from multiple sources ( Harris and Frank, 2014; Holmboe *et al.,* 2011; Dath and Iobst, 2010). Experts recommend a greater focus on faculty skill development for these tasks ( Harris and Frank, 2014; Holmboe *et al.,* 2011; Dath and Iobst, 2010). In particular, teachers working within this model need FD with regard to knowledge of competency-based education, facility in teaching within a CBME system, and new ways to assess and provide feedback to learners in a CBME context ( Harris and Frank, 2014; Holmboe *et al.,* 2010; Fraser *et al.,* 2016). One potential challenge encountered when implementing CBME includes poor faculty attendance at events ( Holmboe *et al.,* 2010; Fraser *et al.,* 2016). Suggestions for improving engagement include faculty incentives, making sessions easily accessible (e.g. online modules) and supporting the development of local CBME champions ( Holmboe *et al.,* 2011; Fraser *et al.,* 2016).

Considering what is currently known about FD for CBME in the literature, the authors wanted to learn more about the local landscape of FD. The overall aim of this study was to harness the collective expertise and opinion of education leaders at UofT to determine their experience and needs with regards to FD for CBME.

## Methods

An online survey was developed to explore the experiences of program directors and residency education leaders for programs currently in or transitioning to CBME. The survey design was informed by the Best Practices in Evaluation and Assessment (BPEA) Working Group. The working group suggested that the survey draw attention to the local context (i.e. university, program, department and site) and focus on change management principles, processes and practices are important factors that influence the successful implementation of CBME ( Takahashi *et al.,* 2017).The survey was developed and pilot tested through an iterative and collaborative process by the authors (SGT, GS) and a small group of colleagues with expertise in FD, medical education and survey design. A combination of multiple choice and open-ended questions were used. The survey was developed using Qualtrics and distributed via email to 168 faculty members. After the initial survey was sent in September 2018, four additional survey reminders were sent to potential respondents and the survey was closed in October 2018. Demographic information was also collected to inform subgroup analysis by program type (e.g. Family Medicine and Royal College).

Descriptive statistics were determined for each multiple-choice question and thematic analysis was applied to the qualitative data. The survey responses to multiple choice questions were summarized using frequency and cross tabulations performed using IBM® SPSS®. The analysis then focused on characterising the qualitative responses to the open-ended questions. One author reviewed the qualitative data (SGT) to identify response categories by grouping related responses into identified themes which was reviewed and refined by a second author (GS). Iterative review was done to reach consensus by all authors for the final categorization of responses. Ethics approval for this study was obtained from the University of Toronto Health Sciences Research Ethics Board.

## Results/Analysis

See Table 1 for selected results. The overall survey response rate was 44.6%. Family Medicine faculty made-up 46.7% of respondents, followed by Medicine at 18.3%. 56.7% of respondents were Program Directors and 26.7% were FD leads. Approximately 80% of respondents had programs that had already launched to CBME. When asked what type of FD activities respondents participated in, most attended workshops with small group activities (98%), accessed website/online resources (94%) and reviewed newsletter updates (87%). The least commonly attended activities included webinars (29.6%), reflective exercises (5.6%) and online, self-instructional modules (3.7%).

**Table 1:** Summary of Key Results

Optimal Timing for FD prior to CBME/CBD Implementation	Responses # (%)
6-12 months prior	23 (45.1%)
More than 1 year prior	21 (41.2%)
Less than 6 months prior	4 (7.8%)
Other	3 (5.9%)
**# of Respondents**	**51 (100%)**
Requested CBME Topics* *Respondents were able to indicate more than one response	**Responses** # (%)
Implementing CBME/CBD	19 (44.2%)
Assessment tools	17 (39.5%)
Competence Committees	15 (34.9%)
Feedback and coaching	15 (34.9%)
EPA assessments (incl how ‘entrustment’ is different)	14 (32.6%)
Online assessment tool use	14 (32.6%)
CBME/CBD update	12 (27.9%)
Assessment models and design	11 (25.6%)
Learner handover	10 (23.3%)
Educational models and design	8 (18.6%)
Change management	7 (16.3%)
Other	2 (4.7%)
**# of Respondents**	**43** **(100%)**
Required FD Supports* *Respondents were able to indicate more than one response	**Responses** # (%)
Administrative support	25 (54.3%)
Support with faculty development program design	25 (54.3%)
Support with faculty development program evaluation	22 (47.8%)
Technical support with online resource development	23 (50.0%)
Current level of resources and support adequate	8 (17.4%)
Other	4 (8.7%)
**# of Respondents**	**46** **(100%)**
Barriers to FD* *Respondents were able to indicate more than one response	**Responses** # (%)
Lack of time amongst faculty	41 (87.2%)
Lack of interest amongst faculty	19 (40.4%)
Lack of administrative support	18 (38.3%)
Lack of expertise in faculty development program design	17 (36.2%)
Lack of experience in faculty development program evaluation	13 (27.7%)
Other barriers	5 (10.6%)
No barriers identified	3 (6.4%)
**# of Respondents**	**47** **(100%)**
Most Helpful CBME FD Activities* *Respondents were able to indicate more than one response	**Responses** # (%)
Small Groups	10 (32.3%)
Rounds	5 (16.1%)
Workshops	5 (16.1%)
Retreats	3 (9.7%)
Online	2 (6.5%)
Combination Model	2 (6.5%)
Other	4 (12%)
**# of Respondents**	**31** **(100%)**

When asked what CBME-related topics the respondents planned on offering in their programs in the upcoming year, the top three subjects were Entrustable Professional Activities (69%), feedback and coaching (67%) and CBME/CBD update (60%). The least likely FD topics respondents were planning included learner handover (11%), change management (9%) and educational models and design (9%).

The top FD topics survey respondents would like to learn more about include implementing CBME, assessment tools, feedback and coaching and competence committees. The most effective FD activity identified by survey respondents was a small group format while the most helpful FD topic was coaching and feedback. The least effective FD activity was online modalities and resources.

The majority of programs felt that the ideal timing for CBME implementation was 6-12 months prior to implementation (45%); however, a large proportion (41%) also felt that FD should begin more than 1 year prior to CBME implementation. Survey respondents felt that the most common supports needed include administrative and technical support for program design/evaluation.

The main perceived barrier to FD was lack of time amongst faculty (87%), followed by lack of administrative support and lack of expertise in FD program design. 81% of faculty respondents did not survey their own faculty at the program level regarding CBME FD needs.

## Discussion

This survey is the first of its kind to evaluate the FD needs and experiences of postgraduate programs broadly at UofT. The data helped Postgraduate Medical Education (PGME) and the Centre for Faculty Development (CFD) at UofT in planning strategically for implementing competency based curricular reforms. The results have also informed the need for additional resources and enabled focused FD on barriers and needs. For example, this survey helped support the development of a feedback and coaching series along with a FD planning tool as a program resource. The survey data was further used to leverage resources for content development.

In terms of lessons learned, the authors found it interesting that, despite the convenience and flexibility of online modalities for FD, the respondents identified small group, interactive sessions as the most successful FD format, while also noting that online modalities were perceived as least effective. The narrative survey comments may help shed some light on this finding. One respondent stated that “faculty need the time and space to learn about CBME... the site-based approach... is best to transfer skills and knowledge on a practical level.” The survey results revealed that one size does not fit all when it comes to the format of the FD activities, but also when it comes to topics of interest. Sessions on coaching and feedback were noted as most the helpful FD topics presented. In particular, respondents found this topic “most valuable for ‘frontline’ teachers.”

When it came to the ideal time for FD related to CBME implementation, most respondents felt that at least 6-12 months of lead time were needed. Respondents felt that faculty needed “adequate time for familiarity with CBD, but not so long that faculty feels it is irrelevant/forgotten.” Those who felt that greater than 1 year of lead time is needed commented that “change management is a long term process” and that “faculty members need repetition”.

A surprising finding was that 81% of respondents had not surveyed their local faculty about their FD needs with respect to CBME, especially in light of the importance that the local context plays in the design and implementation of FD activities. ^9^ Although this survey has helped to inform the design of FD activities at the PGME and CFD level, the authors feel that this does not replace the need for program or department level feedback.

### Limitations

Although the overall survey response rate was acceptable, the small number of respondents within each sub-group did not allow for statistical comparisons between sets.

## Conclusion

### Future Directions

This data will help inform future offerings. For example, the development of FD activities by PGME and the CFD that include peer mentoring and coaching, reflective exercises, change management or role-play with feedback may be of benefit to programs that are not prioritizing these topics currently. Furthermore, longitudinal follow-up of the programs surveyed may be helpful to monitor changes in FD gaps and needs over time.

The authors suggest that all FD should be data driven to spur content development as one size does not fit all. It is clear that FD should be centred on the needs of its learners. These needs will vary and may focus on content development, tool development, along with resources and supports to navigate the systems issues and barriers that can undermine FD for CBME.

## Take Home Messages

• Faculty Development (FD) will be a key component to supporting successful implementation of competency-based medical education (CBME).
• Survey results indicate small group format as the most effective FD activity.
• Top perceived FD topics include: implementing CBME, assessment tools, feedback and coaching along with competence comittees.
• Majority of programs felt CBME implementation 6-12 months prior implemenation as a requirement was the ideal timing.
• The main perceived barrier to FD was lack of time amongst faculty.

## Notes On Contributors

Dr. Giovanna Sirianni, MD, CCFP(PC), FCFP, MScCH

Enhanced Skills Program Director, Department of Family and Community Medicine

Faculty Development Lead, Division of Palliative Care, Department of Family and Community Medicine

Workplace Based Assessment Lead, MD Program, Temerty Faculty of Medicine

Ms. Shantel Walcott, MSc

Quality Assurance Review and Program Evaluation Officer,

Postgraduate Medical Education,

Temerty Faculty of Medicine, University of Toronto

Dr. Susan Glover Takahashi, PhD

Director, Education & Research, Postgraduate Medical Education

Lead, CBME Faculty Development

Wilson Centre Cross-Appointed Researcher

Associate Professor, Department of Family and Community Medicine

Associate Professor, Dalla Lana School of Public Health

Temerty Faculty of Medicine, University of Toronto

ORCiD: https://orcid.org/0000-0003-0722-7876

## References

[ref1] Dath D and Iobst W. (2010) The importance of faculty development in the transition to competency-based medical education. Medical Teacher. 32(8), pp.683–686. 10.3109/0142159X.2010.500710 20662581

[ref2] Fraser A. B. Stodel E. J. Jee R. Dubois D. A. (2016) Preparing anesthesiology faculty for competency-based medical education. Canadian Journal Anesthesia/Journal canadien d’anesth é sie. 63, pp.1364–1373. 10.1007/s12630-016-0739-2 27646528

[ref3] Gutkin C. and Triple C. (2011) New competency-based family medicine curriculum. Canadian Family Physician. 86(7), pp.856.

[ref4] Harris K. A. and Frank J. R. (2014) Competence by Design: Reshaping Canadian Medical Education. Available at: http://www.royalcollege.ca/rcsite/documents/educational-strategy-accreditation/royal-college-competency-by-design-ebook-e.pdf( Accessed: 10 February 2019).

[ref5] Holmboe E. Sherbino J. Long D. M. Swing S. R. (2010) The role of assessment in competency-based medical education. Medical Teacher. 32(8), pp.676–682. 10.3109/0142159X.2010.500704 20662580

[ref6] Holmboe E. S. Ward D. S. Reznick R. J. Katsufrakis P. K. (2011) Faculty Development in Assessment: The Missing Link in Competency-Based Medical Education. Academic Medicine. 86, pp460–467. 10.1097/ACM.0b013e31820cb2a7 21346509

[ref7] Iglar K. Whitehead C. and Glover-Takahashi S. (2013) Competency-based education in family medicine. Medical Teacher. 35(2), pp.115–119. 10.3109/0142159X.2012.733837 23102055

[ref8] Sirianni G. Takahashi S. G. and Myers J. (2020) Taking stock of what is known about faculty development in competency-based medical education: A scoping review paper. Medical Teacher. 42(8), pp.909–915, 10.1080/0142159X.2020.1763285 32450047

[ref9] Takahashi S. G. Ruetalo M. Abrahams C. Whittaker M. (2017) Best Practices in Evaluation and Assessment (BPEA) for Competency-Based Medical Education (CBME) in Residency Education. Available at: http://cbme.postmd.utoronto.ca/wp-content/uploads/2017/09/BPEA-Summary-Report.pdf( Accessed: 01 February 2019).

