# Supplementary material for: Collective Wisdom in Faculty Development for Competency-Based Medical Education: A Needs Assessment and Survey
Source: MedEdPublish (2016). 2021 Jun 3;10:152. [Version 1] doi: 10.15694/mep.2021.000152.1 (PMC10939560; doi:10.15694/mep.2021.000152.1)
Supplement: Peer review history

# Reviewer report: Reviewer response for version 1

**DOI:** 10.21956/mep.19094.r27514

**Published:** 2021-10-13

**Authors:** Haipeng Xiao

**Affiliations:** 1 The First Affiliated Hospital, Sun Yat-sen University

This review has been migrated. The reviewer awarded 3 stars out of 5 The authors presented a need analysis and survey on faculty development for competency-based medical education. Agreeing with the comments by other reviewers, the results did seem largely within expectations, and without the immediate context being more thoroughly described, the transferability of these local findings would be very limited.

Reviewer Expertise:

NA

No decision status is available

# Reviewer report: Reviewer response for version 1

**DOI:** 10.21956/mep.19094.r27516

**Published:** 2021-10-12

**Authors:** Balakrishnan Nair

**Affiliations:** 1 Centre for Medical Professional Development and University of Newcastle

This review has been migrated. The reviewer awarded 3 stars out of 5 Faculty development is an important area that is often done as an after-thought. if we need to implement changes in medical education, we need faculty buy-in and development. This should be well thought out and proactive. This survey highlights the need for faculty development and underlines some key areas. Since this has a small sample size, it may reflect the local preference and practice only.

Reviewer Expertise:

NA

No decision status is available

# Reviewer report: Reviewer response for version 1

**DOI:** 10.21956/mep.19094.r27513

**Published:** 2021-10-11

**Authors:** Ken Masters

**Affiliations:** 1 Sultan Qaboos University

This review has been migrated. The reviewer awarded 3 stars out of 5 The paper deals with gauging needs for faculty development for competency-based medical education using a survey of Faculty with leadership roles in residency education.The survey was conducted in 2018. I see that “online” features only once, quite low down, and only in the context of assessment. It would be interesting to run a follow-up survey now, in light of Covid-19, to see if online activities would have a greater prominence (and if your survey instrument would have asked for it).Some areas that would improve the paper:• Although the survey tool is described, it is not shown. For the paper to be accurately assessed, one should be able to assess the survey form (A reader can gauge most of the questions based on the results, but this is not enough).• While the paper will undoubtedly be of interest to readers at the university, it really should have been better-positioned for a wider audience, by using the Discussion portion to discuss these results in the broader national and international context. Without that context, the paper is a useful internal report, but, unfortunately, does not appear to go further than that.

Reviewer Expertise:

NA

No decision status is available

# Reviewer report: Reviewer response for version 1

**DOI:** 10.21956/mep.19094.r27515

**Published:** 2021-06-10

**Authors:** Leila Niemi-Murola

**Affiliations:** 1 University of Helsinki

This review has been migrated. The reviewer awarded 4 stars out of 5 This is an interesting article focusing on faculty development for competency-based medical education. In my country this will be a challenge, because in our time-based, master-apprentice-system we have not had any formal faculty and programmatic assessment is just a vision for the distant future. However, systematic and rigorous faculty development program will be the first step towards competency-based resident education.The authors made an online survey in order to explore experiences of the faculty working in programs in or transitioning to CBME. The test was developed and pilot tested by the authors. The response rate was acceptable (over 40 %). The results were interesting but not surprising, the most popular faculty development format was a small group activity, the least popular were an online modalities and the most frequent perceived barrier was lack of time.The problems are usually the same for everyone, but the solutions depend on the context. Thus, one size will not fit all. Like the authors point out, a local survey to find out the learning needs, preferred formats and timing of the educational intervention would be helpful.

Reviewer Expertise:

NA

No decision status is available
